# The Status of Dosage Compensation in the Multiple X Chromosomes of the Platypus

**DOI:** 10.1371/journal.pgen.1000140

**Published:** 2008-07-25

**Authors:** Janine E. Deakin, Timothy A. Hore, Edda Koina, Jennifer A. Marshall Graves

**Affiliations:** Research School of Biological Sciences, The Australian National University, Canberra, Australia; Massachusetts General Hospital, Howard Hughes Medical Institute, United States of America

## Abstract

Dosage compensation has been thought to be a ubiquitous property of sex chromosomes that are represented differently in males and females. The expression of most X-borne genes is equalized between XX females and XY males in therian mammals (marsupials and “placentals”) by inactivating one X chromosome in female somatic cells. However, compensation seems not to be strictly required to equalize the expression of most Z-borne genes between ZZ male and ZW female birds. Whether dosage compensation operates in the third mammal lineage, the egg-laying monotremes, is of considerable interest, since the platypus has a complex sex chromosome system in which five X and five Y chromosomes share considerable genetic homology with the chicken ZW sex chromosome pair, but not with therian XY chromosomes. The assignment of genes to four platypus X chromosomes allowed us to examine X dosage compensation in this unique species. Quantitative PCR showed a range of compensation, but SNP analysis of several X-borne genes showed that both alleles are transcribed in a heterozygous female. Transcription of 14 BACs representing 19 X-borne genes was examined by RNA-FISH in female and male fibroblasts. An autosomal control gene was expressed from both alleles in nearly all nuclei, and four pseudoautosomal BACs were usually expressed from both alleles in male as well as female nuclei, showing that their Y loci are active. However, nine X-specific BACs were usually transcribed from only one allele. This suggests that while some genes on the platypus X are not dosage compensated, other genes do show some form of compensation via stochastic transcriptional inhibition, perhaps representing an ancestral system that evolved to be more tightly controlled in placental mammals such as human and mouse.

## Author Summary

Dosage compensation equalizes the expression of genes found on sex chromosomes so that they are equally expressed in females and males. In placental and marsupial mammals, this is accomplished by silencing one of the two X chromosomes in female cells. In birds, dosage compensation seems not to be strictly required to balance the expression of most genes on the Z chromosome between ZZ males and ZW females. Whether dosage compensation exists in the third group of mammals, the egg-laying monotremes, is of considerable interest, particularly since the platypus has five different X and five different Y chromosomes. As part of the platypus genome project, genes have now been assigned to four of the five X chromosomes. We have shown that there is some evidence for dosage compensation, but it is variable between genes. Most interesting are our results showing that there is a difference in the probability of expression for X-specific genes, with about 50% of female cells having two active copies of an X gene while the remainder have only one. This means that, although the platypus has the variable compensation characteristic of birds, it also has some level of inactivation, which is characteristic of dosage compensation in other mammals.

## Introduction

Monotremes are unique mammals that exhibit a mix of reptilian and mammalian features, as they lay eggs, yet have fur and produce milk for their young. Represented only by the fabled platypus and four species of echidna, they are distantly related to humans and other eutherian (‘placental’) mammals, having diverged from therian mammals (eutherians and marsupials) 166 million years ago (MYA) [1].

Monotreme genomes also show a curious mixture of reptilian and mammalian characteristics. They have a smaller genome than therian mammals [2], and their karyotype comprises a few large chromosomes, and many small ones, somewhat reminiscent of chicken macro and microchromosomes. Most curious of all is the sex chromosome system of monotremes. Although monotremes, like other mammals, subscribe to an XY system of male heterogamety, they have multiple X and Y chromosomes [3] which form a multivalent translocation chain during meiosis [4]. Platypus (*Ornithorhynchus anatinus*) have ten sex chromosomes; males have five X chromosomes (X_1_X_2_X_3_X_4_X_5_) and five Y chromosomes (Y_1_Y_2_Y_3_Y_4_Y_5_), and females five pairs of X chromosomes [5]. During male meiosis, X and Y chromosomes pair within terminal pseudoautosomal regions [6], forming a chain of alternating X and Y chromosomes (numbered by their order in the chain X_1_–Y_1_–X_2_–Y_2_–X_3_–Y_3_–X_4_–Y_4_–X_5_–Y_5_) which segregate into five X-bearing (female-determining) and five Y-bearing (male-determining) sperm [7].

The sex chromosomes of therian mammals are remarkably conserved. The X chromosomes of all placental mammals have virtually identical gene contents, and the marsupial X chromosome shares two thirds of the human X, defining it as the ancient X conserved region [8]. The largest platypus X was also thought to share this ancient region [9]. However, comparisons of the gene contents of platypus, human and marsupial sex chromosomes reveal that the ancient region of the therian X is entirely homologous to platypus chromosome 6 [6]. Instead, platypus X chromosomes share considerable homology with the chicken Z chromosome, including *DMRT1*, a dosage-sensitive gene that is a candidate for bird sex determination [6],[10].

The monotreme sex chromosome complex is proposed to have evolved by repeated autosome translocation onto an original bird-like ZW pair [5],[11]. The possession of a chain of nine sex chromosomes by the echidna, seven of which are shared with platypus [12], means that the chain is at least 30 M years old. How a ZW system of female heterogamety was transformed into an XY system of male heterogamety has been vigorously debated [13].

Mammalian Y chromosomes are much smaller and more variable than their X chromosome partners, but share homology within pseudoautosomal regions, and also between coding genes on the X and Y. This supports the theory that heteromorphic sex chromosomes evolved from a pair of homologous autosomes in a mammal ancestor after one member of the pair acquired a sex determining locus, which lead to suppression of recombination and ultimately resulted in differentiation between members of the pair (reviewed in [14],[15]). A similar scenario is proposed for the evolution of the bird Z and W from an ancient autosomal pair [16]. Comparative gene mapping between the mammal X and bird Z [17],[18] shows that they arose from different autosomal pairs.

Although they are non-homologous, the XY of therians and ZW of birds do possess similar general properties. The bird Z, like the mammal X, is highly conserved between species [18], whereas the W is degraded to different extents in different bird groups. Also, the bird Z and the mammal X are large chromosomes carrying many genes, and are well conserved between species, whereas the heterogametic chromosome (W and Y) is small, heterochromatic and varies greatly in size and gene content. The X and Z chromosomes both appear to have sex-biased gene content. For example, the human X chromosome is enriched with genes involved in brain function, sex and reproduction [19]–[21], and in male (but not female) specific genes [22], and the chicken Z is enriched with genes involved in male (but not female) reproduction [23].

Despite these similarities between the mammal XY and the bird ZW sex chromosome systems, the extent to which genes on the X and Z are dosage compensated is remarkably different. X chromosome inactivation overcomes differences in gene dosage between XX females and XY males in therian mammals. In somatic cells of female humans and mice, genes on one X become genetically inactive [24] and transcriptionally silenced [25] early in embryogenesis, a state that is somatically heritable. In marsupials, too, genes on one X chromosome are inactivated [26].

X inactivation mechanisms in eutherians and marsupials differ in a number of important aspects. In somatic cells of eutherians, inactivation is random between maternally and paternally derived X chromosomes, whereas in marsupials only the paternal X is silenced. X inactivation in eutherians is more stable and complete than in marsupials [26], although it was recently discovered that between 5% [27] and 15% [28] of genes on the human X escape inactivation, mostly on the region added recently to the X in the eutherian lineage [28].

At the molecular level, eutherian X inactivation results from a complex process controlled by a master locus (the X inactivation centre XIC), which includes the non-coding *XIST* gene [29],[30]. An array of epigenetic mechanisms, including binding with variant histones [31], histone modifications [32],[33] and differential DNA methylation [34],[35], contribute to the transcriptional silencing of the X-borne genes. An accumulation of LINE1 elements may provide “booster stations” for the propagation of silencing signal along the chromosome [36]. The molecular mechanism of X inactivation seems to be much simpler in marsupials. The region homologous to the XIC in eutherians is disrupted in marsupials and monotremes and no evidence of *XIST* has been found in the regions that juxtapose flanking markers [37]–[39]. *XIST* may have evolved in eutherians from relics of an ancient protein-coding gene [40]. Molecular mechanisms shared between marsupial and eutherian inactivation so far have been limited to late replication [41] and histone underacetylation of the inactive X [42]; DNA methylation does not seem to be involved in marsupial X inactivation [43].

It was suggested that marsupial X inactivation might represent an ancestral form of paternally imprinted X inactivation [26],[44], and this hypothesis is supported by imprinted inactivation in mouse extra-embryonic tissues [45], which, like marsupial X inactivation, is less stable and incomplete, and does not involve DNA methylation [46]. However, unlike marsupials, this imprinted X inactivation in mice requires *Xist* [47],[48]. The XIC, along with an accumulation of LINE1 elements on the X, may control random inactivation in eutherians and its absence correlates to the absence of *XIST* and LINE1 accumulation on the marsupial X [49].

The dosage difference for Z-borne genes between ZZ male and ZW female birds is equally as extreme as for the mammal X. Yet birds do not appear to achieve dosage compensation by silencing one Z chromosome in males, since both alleles can be demonstrated to be active by RNA-FISH and SNP analysis [50],[51]. Quantitative PCR showed that nine of ten Z-borne genes have a male-female ratio close to 1∶1 [52], but in microarrays, 40 zebrafinch and 964 chicken Z-borne genes showed a range of male to female ratios from 2∶1 (∼10% of genes) to 1∶1 (∼10% of genes), with a mode in the middle [53]. In chicken embryos, the mean male to female ratio is 1.4–1.6 for Z-linked genes, consistent with an absence of complete dosage compensation [54]. This incomplete dosage compensation suggests that differences in gene dosage may be critical for only a few genes on the bird Z compared to the mammal X.

The molecular mechanisms behind bird dosage compensation are yet to be elucidated. Differences in male to female ratios between Z linked genes suggest that at least some are regulated at the transcriptional level. A region on the short arm of the Z chromosome containing over 200 copies of a 2.2 kb repetitive sequence called *MHM* (male hypermethylated), is hypermethylated on the Z chromosomes in male embryos, but hypomethylated on the Z in females [55]. *MHM* is transcribed only in females and accumulates as non-coding RNA near the *DMRT1* locus in the nucleus. A higher proportion of genes subject to dosage compensation are clustered in this *MHM* region [56]. This suggests that dosage compensation in birds is via upregulation of gene expression in females, controlled by *MHM* [57].

The platypus presents a fascinating system in which to study dosage compensation. The need for such a system would appear to be acute, since the five X chromosomes of the complex account for 15% of the haploid genome, and are mostly unpaired by the five Y chromosomes, which together account for only 6%, and are at least half heterochromatic. Thus 12% of the genome is subject to 1:2 dosage differences. The homology of the platypus sex chromosomes with the bird Z, and lack of homology with the mammal X, raises questions of whether dosage compensation is incomplete and bird-like, or related to the mammal X inactivation system–or is completely different from both.

There are almost no studies of dosage compensation in monotremes, and none using any molecular techniques. Early studies of replication timing of platypus X_1_ found no asynchronous replication of the unpaired region of this chromosome [58]. This suggests that if the platypus does compensate for gene dosage, it is unlikely to do so by X inactivation. Determining whether the platypus X chromosomes are dosage compensated has previously been difficult in the absence of knowledge of the genes on platypus X chromosomes.

The assignment of genes to four of the five X chromosomes as part of the platypus genome project now presents an opportunity to investigate dosage compensation in this species. We used three different approaches to determine activity of genes located on four of the five platypus X chromosomes, and present evidence of significant transcriptional silencing of platypus X-borne genes.

## Results

We used quantitative real-time RT-PCR, SNPs (Single Nucleotide Polymorphisms) and RNA fluorescence *in situ* hybridization (RNA-FISH) to examine dosage compensation in the platypus. First we gained an overall assessment of the level of dosage compensation by comparing the amounts of transcript from X-specific, autosomal and pseudoautosomal genes in males and females using quantitative real-time RT-PCR. We then identified SNPs within the sequence of X-borne genes to determine if they are expressed from both alleles, or only one, as would be expected from imprinted X inactivation. Finally, we used RNA-FISH to examine the probability of transcription from the two alleles in female and male cells.

### Determination of Male∶Female Expression Ratios by qRT-PCR

We determined male to female gene expression ratios for two autosomal genes and 19 genes on platypus X_1_, X_2_, X_3_ and X_5_, 10 of which are X-specific and nine pseudoautosomal (shared with the Y chromosomes adjacent in the meiotic translocation chain). Genes chosen were from BAC (Bacterial Artificial Chromosome) clones mapped to platypus X chromosomes as part of the genome project [6], as this localization indicated directly whether genes were X-specific or pseudoautosomal. BAC-end sequences from mapped BACs were aligned to the genome to reveal the genomic sequence contained within each BAC. Genes within BACs were identified using the platypus genome Ensembl database (http://www.ensembl.org/Ornithorhynchus_anatinus/index.html) (Oana5.0). The presence of these genes within the BACs was confirmed by PCR and sequencing, and expression of these genes in fibroblasts was determined (Table 1). We used RNA isolated from independently derived primary fibroblast cell lines representing 16 different individuals (eight males and eight females). Expression of these genes was normalized to the expression levels of the housekeeping gene *ACTB,* an autosomal gene located on platypus chromosome 2.

**Table 1:** Genes contained within BACs mapped to X chromosomes as part of genome sequencing project.

BAC	Chromosome	Gene	Expression
636L7	X_1_/Y_1_	*CRIM1*	+
286H10	X_1_/Y_1_	CAMK2A	+
		SLC6A7	+
		CDX1	+
		EN02294§	+
4D21	X_1_	Ox_plat_124086#	+
271I19	X_2_/Y_2_	JARID2	+
		DTNBP1	+
650K19	X_2_/Y_2_	*GMDS*	+
158M16	X_3_	*APC*	+
165F5	X_3_/Y_2_	IRX1	+
830M18	X_5_	EN14997§	+
OaBb_24M14	X_5_	DMRT2	+
		DMRT3*	−
		DMRT1*	−
54B19	X_5_	FBXO10	+
22O3	X_5_	*SHB*	+
752F12	X_5_	*SEMA6A*	+
271G4	X_5_	*SLC1A1*	+
236A5	X_5_	ZNF474	+
		LOX	+

Expression detected in fibroblasts is indicated (+ expressed in fibroblasts; − indicates no detectable expression in fibroblasts). Ensembl gene identifiers have been provided for genes not named in the Ensembl gene build (Jan. 2007). Unless otherwise stated, BAC clones are from the CHORI-236 female platypus BAC library.

# Identifier assigned by the Oxford Functional Genomics group gene build.

*: Expression data from [10]

**§:** These gene names have been abbreviated from the Ensembl gene build designations ENSOANG00000002294, and ENSOANG00000004997.

Male to female ratios were calculated for the normalized data for each gene. The ratio was near 1 for both autosomal control genes (*G6PD* and *HPRT1*) on platypus chromosome 6. We also measured expression levels for nine pseudoautosomal genes with copies on X and Y. The expression ratios of seven genes were high (0.86–1.49), indicating that the Y-borne, as well as the X-borne, alleles are active. However, two pseudoautosomal genes (*CDX1* and *GMDS*) had ratios of about 0.5, suggesting that the Y locus is not active.

For five of the ten X-specific genes, ratios were high (0.81–0.99), as would be expected if genes were largely or fully compensated. However, for three X-specific genes, the ratio was near 0.5, which would be expected if the genes were not compensated between XY males and XX females. Two genes had intermediate ratios (∼0.7), suggesting partial dosage compensation (Table 2). Statistical tests of the null hypothesis that there is no difference in expression levels between males and females, were compromised by the high variability between individuals, which resulted in *p*-values supporting the null hypothesis (*p* = 0.05) for all X-specific genes. This variation could not be attributed to particular cell lines consistently showing higher or lower expression for the different genes tested (see Figure S1). The trend towards a higher level of expression in females than in males for X-specific genes suggests that different genes may be incompletely compensated to different extents.

**Table 2:** Male∶female ratio for expression of platypus X genes in fibroblast cells normalized to the autosomal *ACTB* housekeeping gene.

Gene	Chromosome	Male∶Female Ratio	*p*-value
Autosomal			
*G6PD*	6	1.12	0.21
*HPRT1*	6	0.97	0.96
*Pseudoautosomal*			
*CRIM1*	X_1_/Y_1_	1.15	0.50
*CAMK2A*	X_1_/Y_1_	1.39	0.11
*CDX1*	X_1_/Y_1_	0.50	0.01
EN02294	X_1_/Y_1_	1.48	0.57
*SLC6A7*	X_1_/Y_1_	1.49	0.06
*DTNBP1*	X_2_/Y_2_	0.86	0.47
*JARID2*	X_2_/Y_2_	0.93	0.88
*GMDS*	X_2_/Y_2_	0.48	0.05
*IRX1*	X_3_/Y_2_	0.98	0.85
X-specific			
Ox_plat_124086	X_1_	0.91	0.45
*APC*	X_3_	0.85	0.76
*SHB*	X_5_	0.81	0.43
*LOX*	X_5_	0.94	0.88
EN14997	X_5_	0.71	0.18
*FBXO10*	X_5_	0.73	0.32
*SLC1A1*	X_5_	0.36	0.07
*ZNF474*	X_5_	0.99	0.69
*DMRT2*	X_5_	0.49	0.10
*SEMA6A*	X_5_	0.55	0.14

### SNP Identification and Expression

We used a bioinformatics approach to identify SNPs in genes on four of the five platypus X chromosomes (details in Materials and Methods). We searched the Ensembl database for exonic sequence from predicted genes on platypus chromosomes X_1_, X_2_, X_3_ and X_5_ and compared these to platypus whole genome traces. Within these alignments we searched for single nucleotide mismatches appearing more than once at the same site. Possible SNPs were found in the platypus genome sequence within 57 genes on platypus chromosomes X_1_ (29), X_2_ (6), X_3_ (6) and X_5_ (16). We validated a subset of these SNPs by sequencing PCR products derived from genomic DNA isolated from the same female animal (“Glennie”) used for the genome sequencing project and tested expression of these genes in fibroblast RNA isolated from this same individual. Of ten genes tested, seven were found to be expressed in fibroblasts (ss76901227–ss76901236) (Table 3).

**Table 3:** Genes with SNPs, identified from the genome sequence and validated by PCR and sequencing.

Gene	Chromosome	SNP	Expressed in Fibroblasts
*CCNG1*	X_1_/Y_1_	C/T	+
*GABRB2*	X_1_/Y_1_	C/A	+
*SYNPO*	X_1_/Y_1_	C/T	+
*GMDS*	X_2_/Y_2_	C/T	+
*ADAMTS16*	X_3_	C/T	−
*FRMPD1*	X_5_	C/T	−
*ACO1*	X_5_	G/T	−
*FBXO10*	X_5_	A/C	+
EN14997	X_5_	G/T	+
*SHB*	X_5_	A/G	+

+ indicates expression detected in fibroblasts; − indicates no detectable expression in fibroblasts.

BAC clones for these seven potentially X-specific SNP-containing genes were isolated, by using sequence up to 100 kb either side of the gene to search the platypus trace archive for BAC-end sequences. We confirmed that BACs contained the gene(s) of interest by PCR and direct sequencing. BACs were mapped by DNA-FISH to male metaphase chromosomes to confirm their location on an X and determine whether they have Y homologues (data not shown). Three genes with validated SNPs on X_1_ were found to be pseudoautosomal, and based on genome assembly co-ordinates, all other unvalidated X_1_ SNPs are predicted to likewise fall within the pseudoautosomal region. Similarly, the SNP on X_2_ was shown to have a homologue on Y_2_ by FISH. However, the three X_5_ genes containing SNPs are X-specific.

**Figure 1:**
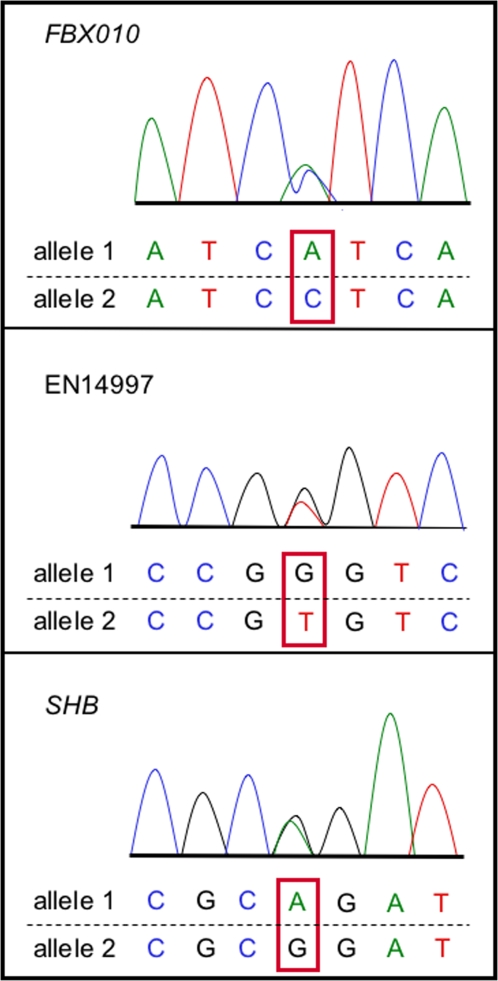
Biallelic expression of three X-specific genes. SNPs (marked by boxes) were identified in the genome sequence demonstrated by sequencing fibroblast cDNA from the sequenced animal (“Glennie”).

Sequencing of X-specific SNPs revealed that all genes were biallelically expressed (Figure 1), as were the pseudoautosomal SNPs (data not shown). Allele specific real-time PCR was used to determine if alleles were expressed to the same extent for the pseudoautosomal gene *GMDS* and the X specific genes. No significant difference from a 1∶1 ratio was observed, implying the absence of imprinting (Table 4 and Figure S2). Biallelic expression with equivalent expression from alternate alleles for the three X-specific genes eliminates the possibility that genes on platypus X_5_ are subjected to complete paternal inactivation (as is observed in marsupials), and directed our approaches to examining the probability of transcription from the two loci by RNA-FISH.

**Table 4:** Relative allele expression determined by allele-specific real-time RT-PCR.

Gene	Allele A	Allele B
*FBXO10* (A/C)	0.47	0.53
EN14997 (G/T)	0.51	0.49
*SHB* (A/G)	0.50	0.50
*GMDS* (C/T)	0.52	0.48

### RNA-FISH Detection of Primary Transcripts

RNA-FISH detects the sites of primary transcription in interphase cells by hybridization with large intronic sequences that are spliced from cytoplasmic mRNA. Thus large genomic probes were required for the genes of interest.

BAC clones mapped to platypus X chromosomes as part of the genome project and found to contain genes expressed in fibroblast, were used for RNA-FISH experiments (Table 1). These included the four clones discussed above (one from X_2_ and three from X_5_). We also included BAC OaBb_24M14 (GenBank Accession No. AC152941) containing *DMRT2,* which had been fully sequenced previously and whose expression had been confirmed in fibroblast cell lines [10]. A BAC containing the *HPRT1* gene located on chromosome 6, OaBb_405M2 (GenBank Accession No. AC148426), was used as an autosomal control. *HPRT1* was detected in the platypus fibroblast EST library sequenced as part of the genome project (GenBank Accession No. EG341684). The 14 BACs together contained 19 genes; two pseudoautosomal BACs contained four and two genes respectively and one X-specific BAC contained two genes (Table S1).

**Figure 2:**
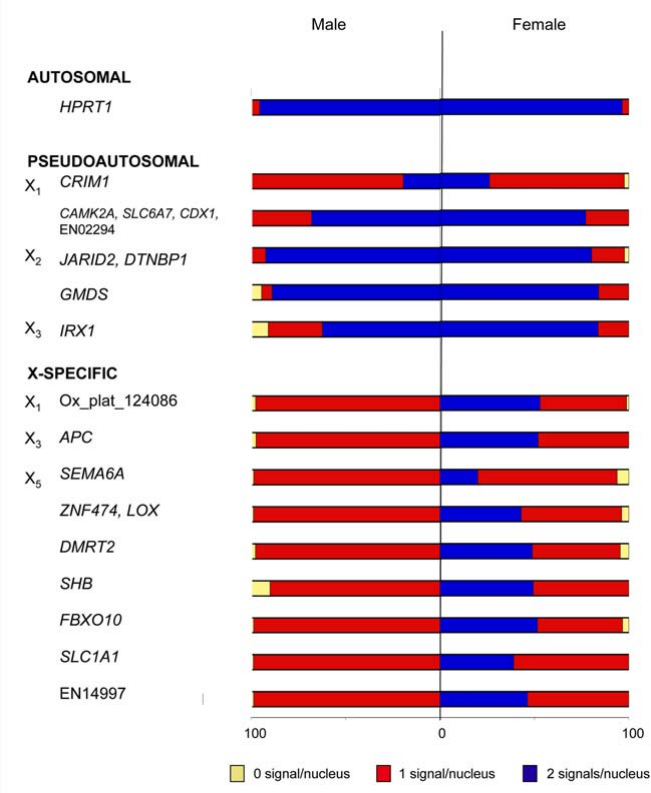
Summary of RNA-FISH results in platypus cells. Frequency of cells in which transcription of no (yellow), one (red) or two (blue) alleles is detected by RNA-FISH in male and female interphase nuclei. Autosomal control, pseudoautosomal and X-specific genes are grouped, with a distinct difference observed between the X-specific genes and the autosomal and pseudoautosomal genes.

Transcription of the 14 BACs described above was initially examined by RNA-FISH in female and male fibroblasts (Figure 2). As a control, RNA-FISH was followed by DNA-FISH to ensure that RNA signals were located near one (X-specific genes in males) or both of the alleles (X-specific genes in females, autosomal and pseudoautosomal genes). Only those cells with two DNA-FISH signals per nucleus (or one signal for X-specific genes in males) were included in analysis. Data from the male RNA-FISH experiments was used to determine the efficiency of detection for each gene which was then used to extrapolate the expected percent of nuclei with biallelic expression in females, which is expected if there is no X inactivation (Table 5 - refer to Table S2 for complete RNA-FISH dataset).

**Table 5:** Expected vs observed frequency of nuclei with biallelic expression in females.

	Efficiency (p)	Female Biallelic Frequency		
		Expected %	Observed %	*P*-value
Autosomal				
*HPRT1*	0.98	96	97	0.96
Pseudoautosomal				
*CRIM1*	0.60	36	26	<0.01
*CAMK2A, SLC6A7, CDX1,* EN02294	0.84	71	77	0.16
*JARID2, DNTBP1*	0.96	92	80	<0.01
*GMDS*	0.92	85	84	0.70
*IRX1*	0.77	59	83	<0.01
X-specific				
Ox_plat_124086	0.98	96	53	<0.01
*APC*	0.97	94	52	<0.01
*SEMA6A*	0.99	98	20	<0.01
*ZNF474, LOX*	0.99	98	43	<0.01
*DMRT2*	0.98	96	51	<0.01
*SHB*	0.90	81	49	<0.01
*FBXO10*	0.99	98	52	<0.01
*SLC1A1*	0.99	98	39	<0.01
EN14997	0.99	98	46	<0.01

Efficiency (p) of RNA-FISH hybridisation was determined from the results obtained in male fibroblasts and extrapolated to determine the expected frequency of nuclei with two signals, one signal and no signal per cell using the formula p^2^+2pq+q^2^ = 1, where p^2^ is the number of nuclei with two signals, 2pq represents nuclei with one signal and q^2^ is the number with no signal. *P*-values were determined by a χ^2^ test with 2 degrees of freedom.

**Figure 3:**
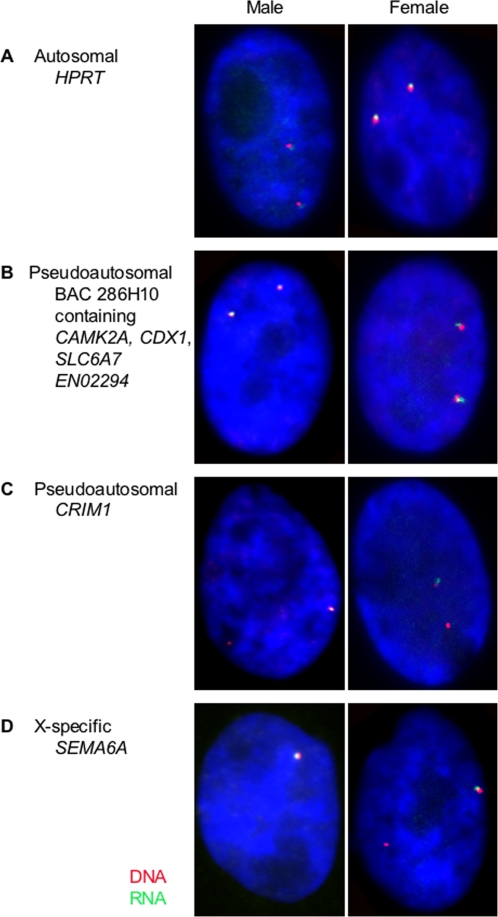
Co-localization of transcripts (RNA - green) and their corresponding gene loci (DNA - red). (A) The autosomal control *HPRT1* is expressed from both loci in both sexes since two signals are detected for both RNA and DNA-FISH in both males and females. (B) Pseudoautosomal BAC 286H10 is expressed from both X chromosomes in females and the X and Y in males, since two signals are detected for both RNA and DNA-FISH in males and females. (C) Pseudoautosomal *CRIM1* located on X_1_ is expressed from only one X in females and only one of the X and Y alleles in males, since two DNA signals but only one RNA signal is detected in both males and females. (D) X-specific *SEMA6A* located on X_5_ is expressed from only one of the two X chromosomes in females, as well as from the single X in males, showing one RNA and DNA signal in males but two DNA signals and only one RNA signal in females.

*HPRT1*, an autosomal control gene located on chromosome 6, was expressed from both alleles in 96–97% of nuclei (Figure 3A). Genes within four pseudoautosomal BACs on X_1_, X_2_ (including *GMDS*) and X_3_ were also expressed from both alleles in most female nuclei (77–84%), as well as in most male nuclei (62–92%), showing that the Y, as well as the X, alleles are active (Figure 3B). Two pseudoautosomal BACs used for RNA-FISH contain more than one gene, so it remains possible that not all genes within these BACs have an active Y copy. We obtained quite different results from the BAC containing *CRIM1*, a X_1_-Y_1_ pseudoautosomal gene which was expressed from only one allele in most male (81%) and female cells (71%) (Figure 3C). Except for this locus, we conclude that for the pseudoautosomal loci we tested, both X alleles are active in females, and both X and Y alleles are active in males.

We then tested transcription from nine X-specific BACs on platypus X_1_, X_3_ and X_5_. Transcription from both alleles was observed on average in only 45% of nuclei (Figure 3D). Different genes showed a range of transcription of both alleles, from 20% (*SEMA6A*) to 53% (*Ox_plat_124086*). These X-specific genes were therefore expressed very differently from the autosomal and pseudoautosomal genes, and significantly different to that expected for biallelic expression, indicating some level of transcriptional inactivation for these genes.

**Figure 4:**
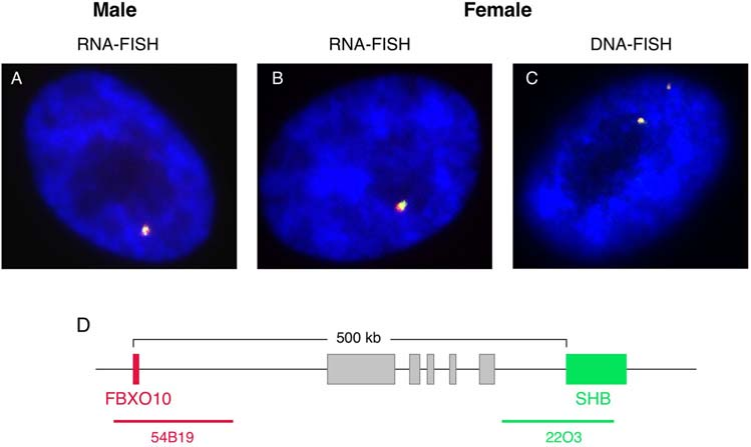
Two-colour RNA-FISH of neighbouring genes *FBXO10* (red) and *SHB* (green). (A) Male nucleus expresses both genes from the single X. (B) Female nucleus expresses both genes from the same single X chromosome. (C) Female DNA-FISH showing that loci are located together. (D) Diagram depicting the region and the location of BACs used for RNA-FISH. Grey boxes indicate genes located between these two BACs.

Two colour RNA FISH was performed with genes *FBXO10* and *SHB*, located within 500 kb of each other. Co-location of the two RNA signals showed the same X in all of the 51% of cells expressing from only one allele. (Figure 4). A few cells (12%) displayed biallelic expression from *SHB* with monoallelic expression of *FBXO10*, and in 37% of nuclei, both genes were expressed from both alleles. As a control, this experiment was performed on male nuclei showing that RNA-FISH signals co-located in all nuclei in which genes were expressed. This experiment was carried out only for two genes lying close together, as results from genes situated further apart (and hence with a gap between signals expressed from the same chromosome) would make results from cells expressing only one of each gene, difficult to interpret.

RNA-FISH results were validated for a subset of genes (*HPRT1, CRIM1, GMDS, SEMA6A* and *DMRT2*) on four other independently derived primary fibroblast cell lines from different individuals (one male and three females). Results for each cell line are shown in Table S3. As observed (Figure 2), the autosomal gene *HPRT* was expressed from both alleles in most nuclei (88% male and 83–90% female), as was the pseudoautosomal gene *GMDS* (86%, 85–90%). The pseudoautosomal gene *CRIM1,* as before, was expressed from both X chromosomes in only 24–56% of female nuclei and X and Y in only 24% of male nuclei. As observed (Figure 2), both X-specific genes (*DMRT2* and *SEMA6A*) were expressed from the single X in 99% of male nuclei, and both X chromosomes in half of female nuclei (45–60% and 38–43% respectively). Although there was some variation between individuals, overall results were similar between all six cell lines tested in this study. Statistical analysis revealed that only the two X-specific genes had a significant difference between the males and females for the number of nuclei expressing only one allele (*p* = 0.0006 and 0.0008 respectively).

## Discussion

The very large proportion of the genome (∼12%) that is X-specific in the platypus, and the homology of the multiple platypus X chromosomes to the chicken Z but not the therian X chromosome, makes them a most interesting species for exploring the origins of dosage compensation in mammals.

We therefore tested the transcription of genes on platypus X_1_, X_2,_ X_3_ and X_5_ in order to search for evidence of random X inactivation (as in eutherian mammals), paternally imprinted X inactivation (as in marsupials), or incomplete and variable dosage compensation (as in chickens). Random inactivation would be manifested as dosage equality between males and females, expression from both SNP variants overall but only a single allele per nucleus detected by RNA-FISH. Paternal inactivation would be manifest by dosage equality, but expression of only one SNP variant, and only one allele per nucleus would be detected by RNA-FISH. Bird-like incomplete dosage compensation would be manifest as a wide range of dosage relationships between males and females, expression of both SNP variants and expression from both alleles in each nucleus.

Our results are not strictly consistent with any of the above predictions. Quantitative RT-PCR showed male∶female expression ratios near 0.5 or 1.0 for different genes, although both SNP alleles were expressed for all genes at an equal level in a heterozygote. Our examination of transcription of X-specific platypus genes by RNA-FISH revealed that about half of female cells expressed only one allele. The RNA-FISH results showed a clear difference between the transcription of X-specific loci compared with pseudoautosomal and autosomal loci.

These data imply that genes from platypus X-specific regions show some form of compensation via transcriptional inhibition, as for mammals, but this is incomplete and variable between genes. Our demonstration that genes were expressed equally from both alleles suggests that paternal inactivation and imprinted partial expression is unlikely. Our demonstration that both alleles are expressed in about half the nuclei rules out complete X inactivation (random or imprinted), as is also seen for many partially escaping genes in eutherians and marsupials.

The variability in overall expression between different X-borne genes resembles the range of expression of genes on the bird Z in males and females that indicates a more relaxed, or more variable, dosage compensation system [53]. Biallelic expression of Z-borne genes was also found by examining expression of different alleles of two genes from fibroblast cultures established from single cells [51]. These results taken together suggest that bird dosage compensation is partial and differs between genes on the Z.

Thus dosage compensation of X-borne genes occurs to some extent in the platypus, and has features of both bird-like and mammal-like sex chromosome dosage compensation.

### Is Partial Inactivation Ancestral?

Together, our findings have parallels in observations of some genes on the marsupial X and the mouse X in extra-embyronic tissues, whose paternal alleles are partially inactive, or “escaper” genes on the recently added region of the human and mouse X, which are partially expressed from the inactive X.

The observations of partial inactivation in all three major mammalian lineages suggests that partial inactivation observed here in platypus represents a basic form of mammalian X inactivation, which has come under tighter control during therian evolution, ultimately resulting in the highly stable and complex form of inactivation typical of most eutherian X-borne genes.

Partial inactivation has been documented for two marsupial genes (out of a total of five) in some tissues. PGK1 isozyme variants showed strong expression from the maternal allele and weaker expression from the paternal allele in cells from heterozygous female kangaroos, even in single clones [59], and *G6PD* from hybrid marsupials showed a heteropolymer band, diagnostic of expression from both alleles in a single cell [60]. Differences between species, tissues and even between genes make it difficult to generalize about the nature of marsupial X inactivation, and these experiments could not distinguish whether partial expression from the paternal X is due to low expression from paternal X chromosomes in every cell, or to a mixture of two X-active and one X-active cells. RNA-FISH was used to show that the tammar wallaby X-borne gene *SLC16A2* was expressed from only one allele in most fibroblast cells [61].

The partial silencing displayed for platypus X-specific genes also has some parallels to genes on the human X that escape inactivation. X inactivation in humans was initially thought to involve all genes on the X chromosome, but in recent years it was found that 5% to as many as 15% of human genes escape inactivation in lymphoblastoid [27] and fibroblast cell lines [28] respectively. Remarkably, transcription of some of these genes in fibroblasts varies between individuals, as seems to be the case for platypus. Partial expression of genes on the inactive X has also been observed in other eutherians, including the mouse, cow and mole [62],[63]. Typically, these escaper genes are fully expressed from the active X and partially expressed from the inactive X [28],[64].

We propose that partial inactivation was the mechanism for compensating differences in gene dosage in an ancestral mammal.

### Partial X Inactivation and the Probability of Transcription

To date, it has been difficult to differentiate between the alternative hypotheses that partial inactivation is due to a lowered rate of transcription in all cells, or from a lowered probability of expression per cell in the population. Ohlsson et al [65] argued that genes transcribed at a low level show a low probability of transcription in the cell population, rather than a uniformly low transcription level. They propose that genomic imprinting and X chromosome inactivation evolved by regulating, not the activity of each locus, but the probability that it is expressed, and making this parent specific [65].

This radical hypothesis is supported by our RNA-FISH data, which show that platypus genes differ in the frequency of nuclei in which one or both alleles are transcribed, giving an overall partial dosage compensation that differs from gene to gene. The data from the bird Z is equivocal; the variability between genes is thought to reflect differences in the rate of transcription, but could equally well reflect differences in the probability that a locus is transcribed. RNA-FISH of five chicken genes shows that most are transcribed from both alleles in most cells [50]; however, the low efficiency of signal detection (about a quarter of nuclei had no signals), and the different tissues used makes this hard to interpret. Efficient RNA-FISH on the chicken Z genes for which we have data in platypus would test the hypothesis that partial inactivation of the Z in male birds operates by altering the probability of transcription, rather than uniformly downregulating transcription.

Our finding that two genes located 500 kb apart are expressed from the same chromosome implies that the stochastic expression of X-specific genes is coordinated in *cis.* Furthermore, a recent study has shown that this type of probabilistic expression is widespread on human autosomes, with their data suggesting that as many as 1000 human genes are subject to stochastic monoallelic expression [66]. Around 80% of these genes also showed some level of biallelic expression. Unlike the hypothesis put forward by Ohlsson et al [64], this type of expression is not limited to those with low levels of expressions.

Is partial expression in therian mammals explained by stochastic expression? Data on partial expression of genes on the paternal X in marsupials are equivocal; the partial expression of the maternal *PGK1* allele in clones, and the fainter paternal isozyme heteropolymer band for *G6PD* are explained equally well by both hypotheses. The few data that would distinguish these hypotheses for escapers on the inactive human X do not conclusively eliminate either hypothesis. Assays of the partially expressed human X-borne gene *CHM (REP1)* in single cells showed that *CHM* was expressed from the inactive X in most (70%) but not all cells from one cell line, and in only seven out of ten hybrid cell lines carrying an inactive X [67]. More recently, a study on dosage compensation in human lymphoblastoid cell lines found that genes escaping X inactivation were not subject to the higher levels of variation found for fibroblast cell lines, suggesting that the expression of the escaper genes is not stochastic but subject to tight regulation [27]. RNA-FISH performed on both fibroblasts and lymphoblastoid cells for these escaper genes would conclusively rule out stochastic expression.

It is important to note the difference in the number of genes in human which escape inactivation between fibroblast cell lines, where 15% of genes are said to escape inactivation [28] and lymphoblastoid cell lines where only 5% of genes escape [27]. Similarly in marsupials, differences have been found in the inactivation status of genes between tissues [26]. Our study has only used fibroblast cell lines due to the difficultly in obtaining tissue samples in large enough sample sizes, as the platypus is listed as a “vulnerable” species. A comparison of results for other tissues may show different results.

Several human X-borne genes that escape from inactivation have a widely expressed Y homologue, and some others have homology to a Y-borne pseudogene that represents a recently inactivated partner on the Y. The Y homologue of an X/Y pair often has a lower level of expression than its partner on the X (reviewed in [68]), similar to the lower level of expression exhibited by alleles on the inactive X in females. However, the presence of a Y homologue does not necessarily negate the need for dosage compensation, as some Y alleles have evidently taken on functions different from those of their X homologue. Nearly all escaper genes are part of the region added to the eutherian X chromosome and only recently recruited to the inactivation system, suggesting that their partial escape from X inactivation correlates with progressive assimilation of genes into the X inactivation systems once the Y paralogue has degenerated.

### Pseudoautosomal Genes and Inactivation

In eutherian mammals, small terminal regions of the X and Y are homologous, and pair and recombine at male meiosis. These pseudoautosomal regions (PARs) are relics of the X added region that have not yet degraded [15]. Genes within the PAR have no need of dosage compensation.

There are two PARs on the human X. PAR1 on the short arm represents a relic of ancient XY homology, and contains genes that are expressed from the Y, and not inactivated on the X [69]. The smaller PAR2 was added very recently to the long arm of the Y from the long arm of the X, but two genes in the region (*SYBL1* and *SPRY3*) are subject to inactivation, not only on the inactive X, but also on the Y [70].

We observed that seven of the nine platypus genes from the pseudoautosomal regions displayed as much or more expression from males than females, as assessed by quantitative RT-PCR, suggesting that they are expressed from Y as well as the X alleles. RNA-FISH of these genes showed that both alleles were expressed in most cells in females (two X alleles) and males (X and Y alleles). Two of these BACs contained multiple genes, so detection of predominantly two signals per cell does not necessarily mean that all genes are active on both chromosomes; however, expression analysis of transcripts from each of these BACs confirms that most of these genes (3/4 in BAC 286H10 and 2/2 BAC 271I19) have active Y homologues. Two pseudoautosomal genes *CDX1* and *GMDS* had male∶female expression ratios near 0.5 but an almost equal probability of expression, suggesting that either both alleles are downregulated in males, or alternatively, the Y allele sequence has sufficiently diverged from that of the X homologue, leaving it unable to be amplified by our primers.

A fifth platypus pseudoautosomal gene showed a completely different expression pattern. *CRIM1* (cysteine rich transmembrane BMP regulator 1), located on platypus X_1_-Y_1_, had equivalent expression in males and females, but was usually expressed from only one allele in both males (81% of nuclei) and females (69%). There are two possible explanations. Firstly, the Y homologue may have evolved a new male-specific function like many genes on the human Y [15], and be testis specific, so silencing of one X in females evolved to equalize expression of the X homologue. Alternatively, inactivation of both X and Y could be equivalent to the silencing of PAR2 genes on the long arm of the human X. *SYBL1* and *SPRY3* undergo silencing on both the X and Y, the product of their evolutionary history as a block transposed from the X (where it was subject to inactivation) to the Y, where it was dosage compensated to match the X [70].

Thus for most pseudoautosomal genes there is no need for dosage compensation on the X because the Y allele is active, and no dosage compensation is observed.

### Is More Tightly Controlled Dosage Compensation Linked to Gene Function?

The chromosome-wide X inactivation in mouse and human has given rise to the expectation that dosage compensation for genes on sex chromosomes is critical for life. However, this does not seem to be the case in birds. Dosage compensation for the 964 genes on the bird Z chromosome extends over a range from complete compensation (∼10% genes) to no compensation (∼10% genes) with most falling between these extremes [53]. This suggests either that the necessity for strict dosage compensation has been over-emphasized, or that genes on the bird Z chromosome are much more tolerant of dosage differences than genes on the therian X [71].

By no means are all genes dosage sensitive [71]. For instance, many protein products, such as enzymes, are controlled at different levels in the cell, so transcriptional control is not essential. For some genes, a dosage difference may even be essential for function; for instance, a 2∶1 dosage of *DMRT1* has been suggested to define male versus female development in birds [72].

One gene that does not display equal expression between males and females and may even be hypertranscribed in females of both platypus and zebrafinch is *SEMA6A,* a gene on platypus X_5_ and the avian Z. From our data, platypus *SEMA6A* appears not be subject to dosage compensation by real-time RT-PCR, yet RNA-FISH results show that it predominantly has only one allele active per cell. In zebrafinch liver, *SEMA6A* is expressed more than two-fold more in females with just one copy than males with two copies [53]. Although these results were obtained from different cell types in the different species, it is intriguing that in both cases there is some evidence of hypertranscription in females.

It is therefore likely that only a minority of genes on the mammalian X really need to be dosage compensated. The difference in the level of control of sex chromosome activity may therefore be a side-effect of the mechanism used for dosage compensation. Eutherian mammals subscribe to a whole-X mechanism in which inactivation spreads along the X. The bird Z, however, seems to have a piecemeal dosage compensation system in which different genes appear to show different levels of compensation, and compensated genes are clustered [56].

The alternative is that the genes on the bird Z and therian X evolved under different selective pressures. We know that the gene content of these chromosomes is different, having originated from two different pairs of autosomes, and we also know that the gene content of sex chromosomes is biased toward sex-specific expression. The human X is enriched for genes involved in brain function, and sex and (particularly male) reproduction [19]–[22]. The chicken Z chromosome gene content is male-biased yet noticeably deficient in female-biased genes [23]. Commenting on the finding that dosage compensation in birds is much less tightly controlled than in therian mammals, Graves and Disteche [71] suggested that expression differences in Z-borne genes between males and females may have been selected for to control sex-specific characters. Since platypus sex chromosomes show considerable homology to the bird Z, the functions of platypus X-borne genes are likely to be equivalent to those on the chicken Z.

Perhaps, then, partial and variable silencing in the platypus dosage compensates some essential genes, leaves some genes uncompensated where dosage differences are essential for sex-specific function, and partially compensates most genes in proportion to their dosage-sensitivity, as is evidently the case for birds.

### Conclusions

We found that genes on the multiple platypus X chromosomes show partial and variable dosage compensation. This is very similar to the partial and variable dosage relationships of genes on the chicken Z chromosome, with which the platypus X chromosomes share considerable homology. However, unlike birds, platypus dosage compensation involves transcription from only one of the two alleles in a proportion of cells and is coordinated at least on a regional level. Transcriptional inhibition is a property shared by X chromosome inactivation in therian mammals. Thus, platypus dosage compensation has features shared with dosage compensation of the bird Z and the mammal X.

## Materials and Methods

### Identification of Expressed Genes within BACs Mapping to the X Chromosomes

BAC-end sequences from CHORI-236 BAC clones (http://bacpac.chori.org), mapped to platypus X chromosomes as part of the genome project, were aligned against the genome sequence. Genes within the genomic region contained between the BAC-end sequences were identified by using the Ensembl database (http://www.ensembl.org/Ornithorhynchus_anatinus/index.html). An additional four BACs were chosen because they span genes with SNPs that were potentially X-specific. These BACs were identified by searching the platypus sequence trace archives containing BAC-end sequence data (http://www.ncbi.nlm.nih.gov/Traces) with genomic sequence from 100 kb up and downstream of the gene of interest.

PCR was performed on the BACs to confirm that the genes predicted to be contained within the BAC were present. The PCR cycling conditions for all primers were as follows: an initial denaturing step of 94°C for 2 min, 30 cycles of 94°C for 30 sec, annealing for 30 sec at the appropriate temperature (Table S4), 72°C for 1 min and a final extension at 72°C for 10 min.

To determine whether genes within BACs were expressed in fibroblasts, total RNA was extracted from female and male fibroblast cell lines using Gene Elute Mammalian Total RNA Miniprep extraction kit (Sigma). RNA was treated with DNA-free (Ambion) to remove any contaminating DNA and Superscript III (Invitrogen) was used to generate cDNA using random hexamers as primers for first strand synthesis. To ensure there was no genomic DNA contamination in the cDNA sample, a RT-negative control was made by excluding the Superscript III enzyme from the first strand synthesis reaction and was used as a negative control in all RT-PCR experiments. Where possible, primers were designed to span introns. Primers, annealing temperatures and product sizes are listed in Table S4. PCR was carried out using the same cycling conditions described above. Each set of primers was tested on female and male RT-positive and RT-negative samples as well as genomic DNA. PCR products were gel purified using a QIAquick Gel Extraction kit (Qiagen) and directly sequenced by AGRF (Brisbane).

### DNA-FISH on Metaphase Chromosomes

For the four BACs not previously mapped, 1 µg of DNA from these BACs was labeled by nick translation with digoxigenin –11-dUTP (Roche Diagnostics), Spectrum-Orange or Spectrum-Green (Vysis). Unincorporated nucleotides were removed from Spectrum-Orange and Spectrum-Green labeled probes using ProbeQuant G50 micro columns (GE Healthcare). Probes were precipitated with 1 µg platypus C_0_t1 DNA and hybridized to male and/or female platypus metaphase chromosomes and fluorescent signals for digoxigenin labeled probes were detected using the protocol described by Alsop et al [73]. A Zeiss Axioplan2 epifluorescence microscope was used to visualize fluorescent signals. Images for DAPI-stained metaphase chromosomes and fluorescent signals were captured on a SPOT RT Monochrome CCD (charge-coupled device) camera (Diagnostic Instruments Inc., Sterling Heights) and merged using IP Lab imaging software (Scanalytics Inc., Fairfax, VA, USA).

### Quantitative Real-Time RT-PCR

Total RNA was extracted from eight different male and eight different female fibroblast (toe web) cell lines (at passage 6 to 8) to represent a total of 16 individuals. First-strand cDNA was synthesized by oligo (dT) priming using Superscript III (Invitrogen). Primers for each gene were designed using the Plexor program (Promega) (Table S4). PCR reactions were carried out using Quantitect SYBR Green PCR kit (Qiagen) according to the manufacturer's instructions. Amplifications were performed and detected in a Rotorgene 3000 cycler (Corbett Research). To determine the detection range, linearity and real-time PCR amplification efficiency for each primer pair, standard curves were calculated over a 10-fold serial dilution of fibroblast cDNA. A series of two-fold serial dilutions were also carried out to confirm the ability of the PCR conditions to detect this level of difference in expression. All dilutions and samples were run in triplicate. Cycling conditions consisted of an initial hold cycle of 95°C for 15 min, 40 cycles of 94°C for 15 sec, annealing at the appropriate temperature listed in Table S4 for 15 sec and extension at 72°C for 20 sec for data acquisition. Melting curves were constructed from 45°C–95°C to confirm the purity of the PCR products and direct sequencing of products was performed to confirm their identity. Relative expression of each gene was determined by normalization to *ACTB* expression using the formula where the ratio of *ACTB* to target = (1+E_Ref_)^CtRef^/(1+E_Target_)^CtTarget^ [74]. Statistical significance was assessed, for the null hypothesis that there was no difference between male and female expression levels, using an unrelated samples 2-tailed *t* test with unequal variance.

### Bioinformatic Prediction of Expressed Single Nucleotide Polymorphisms (SNPs) in Platypus

Exonic sequence from predicted genes on platypus chromosomes X_1_, X_2_, X_3_ and X_5_ were extracted from the Ensembl 46 database, using the Biomart tool (http://www.ensembl.org/biomart/martview). These sequences were compared to the platypus whole genome shotgun sequence traces (“*Ornithorhynchus anatinus* WGS”) deposited on the trace archive at NCBI (http://www.ncbi.nlm.nih.gov/Traces), using MegaBLAST [75]. Potential single-nucleotide polymorphisms (SNPs) were discovered by manually searching within the BLAST output for single nucleotide mismatches occurring in approximately 50% of target traces. The chromatogram files containing a potential SNP were extracted from the trace archive and assembled using Sequencher™ 4.7 (Gene Codes Corporation, Michigan). This assembled sequence (including surrounding intronic sequence) was tested for uniqueness within the platypus genome using BLAT [76] on the UCSC test browser (http://genome-test.cse.ucsc.edu).

### Allele Specific Real-Time PCR

To validate identified SNPs and test expression in fibroblasts, DNA was extracted from the “Glennie” fibroblast cell line using the Dneasy Blood and Tissue kit (Qiagen) and RNA was extracted as described above. First strand synthesis was performed on RNA using the Supercript III First-Strand Synthesis System for RT-PCR kit (Invitrogen) according to manufacturer's instructions. PCR and RT-PCR was carried out using the primers listed in Table S4.

To quantify the expression level of SNPs for three X-specific SNPs and one pseudoautosomal gene, allele-specific real-time PCR was carried out. Allele specific primers were designed with the 3′end base of either the forward or reverse primer corresponding to the specific allele (refer to Table S5 for primer sequences and corresponding annealing temperatures). The different alleles were amplified in separate tubes. Real-time PCR was performed using Quantitect SYBR Green PCR kit (Qiagen) with amplifications performed and detected in a Rotorgene 3000 cycler (Corbett Research). Cycling conditions are the same for those described in the quantitative PCR section with all samples run in triplicate. Genomic DNA for “Glennie” was included as a control since the allele frequency ratio should be 1∶1, permitting allele-specific amplification bias to be detected and corrected. Known homozygous cDNA samples and pooled homozygous samples with varying ratios of each allele (0.2, 0.4, 0.6, 0.8) were included to ensure the technique was sensitive enough to detect small differences. Allele relative expression levels were calculated using the formula: frequency of allele A = 1/(2E^ΔCt^+1) [77], where ΔCt = (A_cDNA_−B_cDNA_)−(A_gDNA_−B_gDNA_) and converted to a ratio of allele A to allele B. PCR products were sequenced to confirm the identity of products.

### RNA/DNA-FISH on Interphase Nuclei

Male and female fibroblast cells (from toe web) were cultured on gelatin-coated coverslips in AminoMax C100 medium (Invitrogen) at 30°C in an atmosphere of 5% CO_2_. Cells on coverslips were washed with PBS, permeabilized for 7 minutes on ice using CSK buffer plus Triton X (100 mM NaCl, 300mM sucrose, 3 mM MgCl_2_, 10 mM PIPES pH 6.8, 2 mm Vanadyl Ribonucleoside Complex (VRC), 0.5% Triton X) and fixed in 3% paraformaldehyde for 10 minutes. Coverslips were dehydrated via a series of ethanol washes (70%, 80%, 95%, 100%), air-dried and denatured. Probes were labeled as described in the DNA-FISH on metaphase chromosomes section. Hybridization buffer (4×SSC, 40% dextran sulphate, 2 mg/ml BSA, 10 mM VRC) was added to each probe. Probes were denatured at 75°C for 7 min and allowed to preanneal for 20 min. 10 µl of probe was added to each coverslip and hybridized overnight in a humid chamber at 37°C. Coverslips were washed in 0.4×SSC with 0.3% Tween 20 at 60°C for 2 minutes followed by a wash in 2×SSC with 0.1% Tween 20 for 1 min at room temperature. Coverslips were fixed in 3% paraformaldehyde for 10 minutes, treated with 0.1 mg/ml RNase for 1 hour at 37°C and subjected to DNA-FISH following the same hybridization protocol described for DNA-FISH on metaphase chromosomes. Nuclei were viewed under a fluorescence microscope in several different focal planes, with 100 nuclei examined for each probe for both males and females.

Efficiency (p) of RNA-FISH hybridisation was determined from the results obtained in male fibroblasts and extrapolated to determine the expected frequency of nuclei with two signals, one signal and no signal per cell using the formula p^2^+2pq+q^2^ = 1, where p^2^ is the number of nuclei with two signals, 2pq (q = 1−p) represents nuclei with one signal and q^2^ is the number with no signal. *P*-values were determined by a χ^2^ test with two degrees of freedom.

Inconsistencies between RNA-FISH results in previous experiments examining transcription have been attributed to the inability to detect weak signals, which could be overcome by, not only using a combination of RNA and DNA-FISH, but also by amplifying the RNA-FISH signal [78]. In order to ensure that the differences between autosomal, pseudoautosomal and X-specific genes were not due to the inability of the technique to detect both transcripts, an experiment where BACs containing *SEMA6A* and *CRIM1* were labeled with either Spectrum Green or Spectrum Orange (Vysis) or with biotin-16-dUTP (Roche Diagnostics) was performed. Biotin-labeled probes were detected with avidin-FITC (Vector Laboratories Inc.), with FITC signals amplified by additional layers of biotinylated anti-avidin (Vector) and avidin-FITC. No differences between direct labeling and biotin labeling followed by amplification were detected.

## Supporting Information

Figure S1 Real-time results for X-specific genes. Each point is a different cell line (shown in the same order in each graph). Male cell lines are shown in blue, female cell lines in red. Expression has been normalised to ACTB.
(0.15 MB PDF)

Figure S2 Allele-specific real-time RT-PCR results for EN14997. Standards for each allele are shown in red or green and “Glennie” cDNA in pink. cDNA from homozygous individual for the opposite allele in each case is in dark grey, showing that the primers do not amplify both alleles. No template control is light grey.
(0.09 MB PDF)

Table S1 Ensembl Identifiers, genome co-ordinates and corresponding location in human and chicken for genes found within BACs used for RNA FISH.
(0.04 MB DOC)

Table S2 RNA-FISH dataset.
(0.03 MB DOC)

Table S3 RNA-FISH results for three additional female and one male cell lines.
(0.03 MB DOC)

Table S4 List of primers used for SNP validation (SNP), confirmation of expression in fibroblasts (Expression), BAC confirmation (BAC) and qRT-PCR.
(0.06 MB DOC)

Table S5 Primers used for allele-specific real-time PCR.
(0.03 MB DOC)
