# Supplementary material for: The Status of Dosage Compensation in the Multiple X Chromosomes of the Platypus
Source: PLoS Genet. 2008 Jul 25;4(7):e1000140. doi: 10.1371/journal.pgen.1000140 (PMC2453332; doi:10.1371/journal.pgen.1000140)

Figure S1: Real-time results for X-specific genes. Each point is a different cell line (shown in the same order in each graph). Male cell lines are shown in blue, female cell lines in red. Expression has been normalised to *ACTB*.

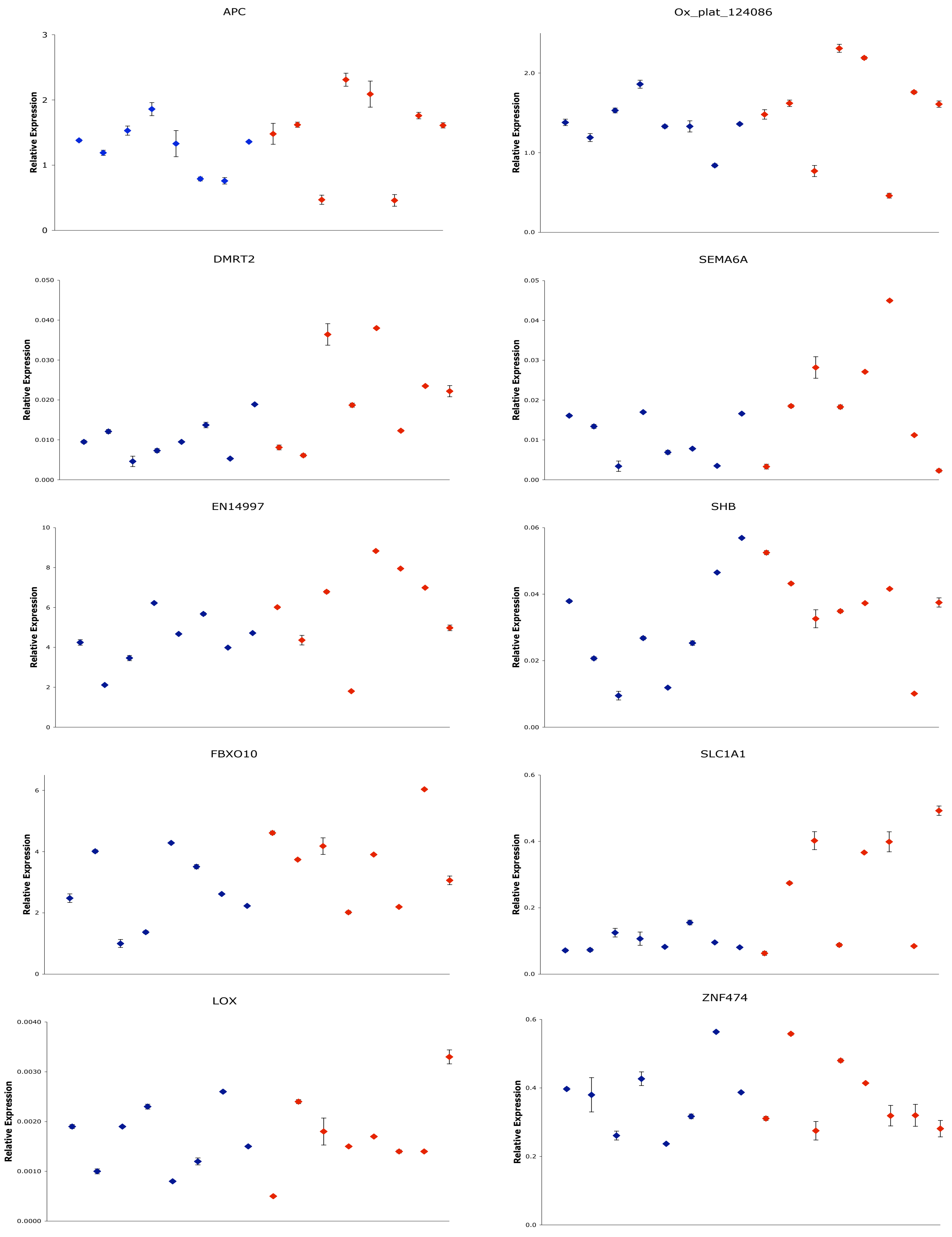

Supplement: Figure S1 — Real-time results for X-specific genes. Each point is a different cell line (shown in the same order in each graph). Male cell lines are shown in blue, female cell lines in red. Expression has been normalised to ACTB. (0.15 MB PDF) [file pgen.1000140.s001.pdf]
