# Supplementary material for: The Status of Dosage Compensation in the Multiple X Chromosomes of the Platypus
Source: PLoS Genet. 2008 Jul 25;4(7):e1000140. doi: 10.1371/journal.pgen.1000140 (PMC2453332; doi:10.1371/journal.pgen.1000140)

Figure S2: Allele-specific real-time RT-PCR results for EN14997. Standards for each allele are shown in red or green and “Glennie” cDNA in pink. cDNA from homozygous individual for the opposite allele in each case is in dark grey, showing that the primers do not amplify both alleles. No template control is light grey.

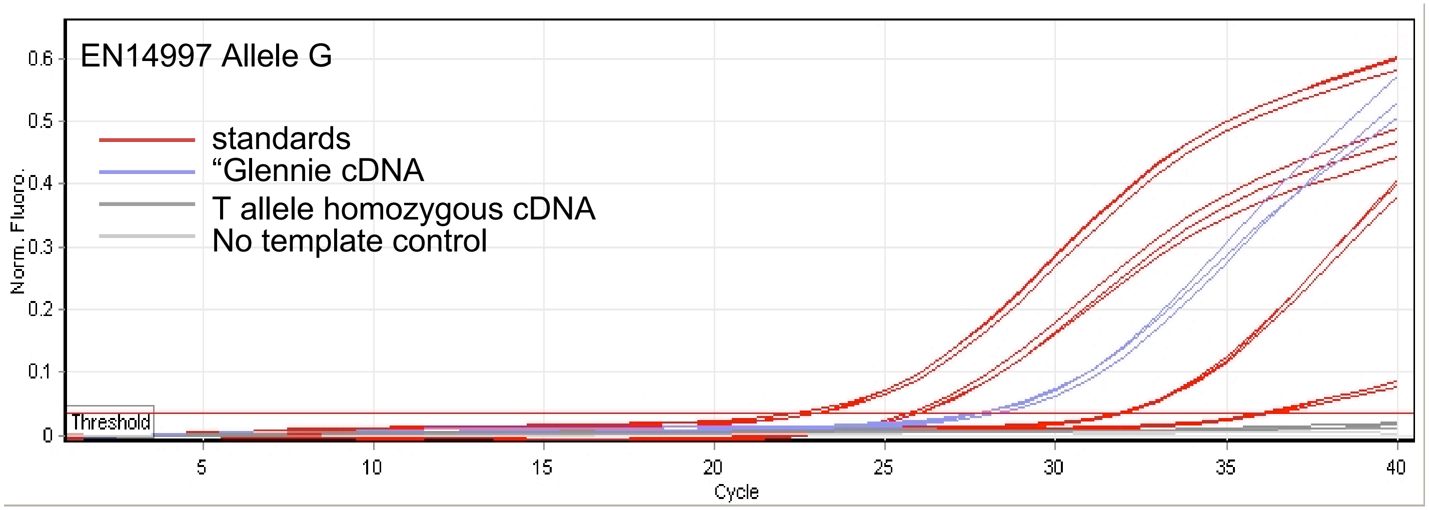

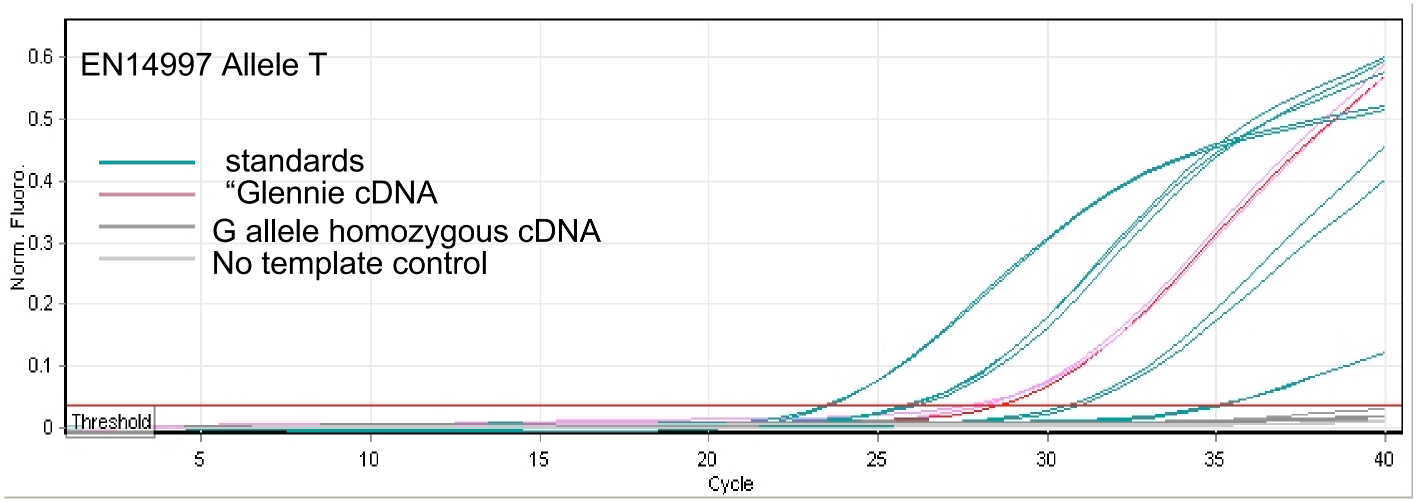

Supplement: Figure S2 — Allele-specific real-time RT-PCR results for EN14997. Standards for each allele are shown in red or green and “Glennie” cDNA in pink. cDNA from homozygous individual for the opposite allele in each case is in dark grey, showing that the primers do not amplify both alleles. No template control is light grey. (0.09 MB PDF) [file pgen.1000140.s002.pdf]
