# Supplementary material for: The Status of Dosage Compensation in the Multiple X Chromosomes of the Platypus
Source: PLoS Genet. 2008 Jul 25;4(7):e1000140. doi: 10.1371/journal.pgen.1000140 (PMC2453332; doi:10.1371/journal.pgen.1000140)
Supplement: Table S1 — Ensembl Identifiers, genome co-ordinates and corresponding location in human and chicken for genes found within BACs used for RNA FISH. (0.04 MB DOC) [file pgen.1000140.s003.doc]

Table S1: Ensembl Identifiers, genome co-ordinates and corresponding location in human and chicken for genes found within BACs used for RNA FISH

| **BAC** | **Chromosome** | **Gene** | **Ensembl or GenBank ID** | **Genome Co-ordinates** | **Human Location** | **Chicken Location** |
| --- | --- | --- | --- | --- | --- | --- |
| CH236_636L7 | X_1_/Y_1_ | *CRIM1* | ENSOANG00000009476 | X1:1234561:1309709 | 2p21 | 3 |
| CH236_286H10 | X_1_/Y_1_ | *CAMK2A*  *SLC6A7*  CDX1  Novel | ENSOANG00000002299  ENSOANG00000002296  ENSOANG00000002295  ENSOANG00000002294 | X1:29054905-29112150  X1:29134237-29146969  X1:29166010-29168118  X1:29208726-29234119 | 5q33.1  5q31-q32  5q31-q33 | 13  13  13 |
| CH236_4D21 | X_1_ | Ox_plat_124086 | EU159185 | X1:41216970-41217076 |  |  |
| CH236_271I19 | X_2_/Y_2_ | *JARID2*  *DTNBP1* | ENSOANG00000011869  ENSOANG00000011868 | Ultra144:725207-830241  Ultra144:833296-909141 | 6p24-p23  6p22.3 | 2  2 |
| CH236_650K19 | X_2_/Y_2_ | *GMDS* | XM_001510089 | X2:4545306 - 5137474 | 6p25 | 2 |
| CH236_158M16 | X_3_ | *APC* | ENSOANG00000000850 | Ultra84:1693846-1739516 | 5q21-q22 | Z |
| CH236_165F05 | X_3_/Y_2_ | IRX1 | ENSOANG00000001749 | X3:3674170-3676215 | 5p15.3 | 2 |
| CH236_830M18 | X_5_ | Novel | ENSOANG00000014997 | X5:1742903-1934516 |  |  |
| OaBb_24M14  (AC152941) | X_5_ | *DMRT2*  DMRT3  DMRT1 | ENSOANG00000008296  ENSOANG00000008295  ENSOANG00000008294 | X5:[3609099-3614291](http://www.ensembl.org/Ornithorhynchus_anatinus/contigview?l=X5:3609099-3614291)  X5:3667914-3681880  X5:3761337-3761615 | 9p24.3  9p24.3  9p24.3 | Z  Z |
| CH236_54B19 | X_5_ | FBXO10 | ENSOANG00000007573 | X5:5194306-5198753 |  |  |
| CH236_22O3 | X_5_ | *SHB* | ENSOANG00000000882 | X5:5693000-5736775 | 9p12 | Z |
| CH236_752F12 | X_5_ | *SEMA6A* | ENSOANG00000008223 | X5:10852802-10915393 | 5q23.1 | Z |
| CH236_271G4 | X_5_ | *SLC1A1* | ENSOANG00000013192 | X5:11420176-11444338 | 9p24 | Z |
| CH236_236A5 | X_5_ | *ZNF474*  *LOX* | ENSOANG00000010045  ENSOANG00000010043 | X5:26857963-26859595  X5:26909367-26916889 | 5q23.1-q23.2  5q23.2 | Z |
| OaBb_405M2  (AC148246) | 6 | *HPRT1* | ENSOANG00000011689 | 6:8425973-8441511 | Xq26.1 | 4 |
