# Supplementary material for: The Status of Dosage Compensation in the Multiple X Chromosomes of the Platypus
Source: PLoS Genet. 2008 Jul 25;4(7):e1000140. doi: 10.1371/journal.pgen.1000140 (PMC2453332; doi:10.1371/journal.pgen.1000140)
Supplement: Table S4 — List of primers used for SNP validation (SNP), confirmation of expression in fibroblasts (Expression), BAC confirmation (BAC) and qRT-PCR. (0.06 MB DOC) [file pgen.1000140.s006.doc]

Table S4: List of primers used for SNP validation (SNP), confirmation of expression in fibroblasts (Expression), BAC confirmation (BAC) and qRT-PCR.

| **Gene** | Primers | **Use** | **Annealing Temp** | **Product size**  **(bp)** |
| --- | --- | --- | --- | --- |
| *ACO1* | F: -AGCAAAGAAAGCTGTAGAAGCTGG  R: TGAGACAGATAAGGCATCACTCCAC | SNP, Expression | 55°C | 123 |
| *ACTB* | F: CTCCTAAGTAAGGGTCTGCTGGAA  R: CCGTCTTTCCCTCCATCGTTG | qRT-PCR | 65°C | 88 |
| *ADAMTS16* | F: AAGTGCTACTGAGGAGTTTCGGTG  R: CATTTGGGGATTGATTCGCTC | SNP, Expression | 55°C | 213 |
| *APC* | F: CGATGGCTTCTTCAGGACAGATTG  R: ACGACTTGATACAGACCCTTCCCG | Expression, qRT-PCR  BAC | 55°C | 143  598 |
| *CAMK2A* | F: ATCTGCCGTCTTCTGAAGCATCC  R: GCCACGATGTCCTCAAACAGCTC  gDNA R:CAAACACAAGGAGACTGTAGACC | Expression, qRT-PCR  BAC | 55°C | 122  582 |
| *CCNG1* | F: CATCTTGGGTGTGTTGGATTGAG  R: GGAGCAACTGGAAAGCTGTAGTAGC | SNP, Expression | 55°C | 198 |
| *CDX1* | F: TACACGGACCATCAGCGTCTTG  R: CACTTTCCTCTCTTTTGCTCTTCG  F: AGGAGTTCCATTACAGCCGCTATA  R: TGCTCTTCGATTCTGGAACCAGA | Expression, BAC  qRT-PCR | 55°C | 156  1911 |
| *CRIM1* | F: CCGCCTCTACTCTGCCAAAATCC  R: CTCTGCTGCCAGGAAAATGTCCC  gDNA R: GGGATAGCGACTGTGTCTGACC  F: GAAGTCTGCCCGCCTCTACTC  R: ATTTGAAGAGGAAGGCTGAAGAGG | Expression  BAC  qRT-PCR | 55°C | 150  472 |
| *DMRT2* | F:CTCCTAAGTAAGGGTCTGCTGGAA  R:CAGGGAAACAGAACCACTTTGAG | qRT-PCR | 65°C | 123 |
| *DTNBP1* | F: tcttcataaaggagccaaggagtg  r: GGTCAGAGTTGCTGTCAAGGATTC  F: CTTGGGCAGCTCTTCATAAAGGAG  R: GACTGTTCTGCTTTTTCTCCCAGT | Expression, BAC  qRT-PCR | 55°C | 175  1432 |
| FBXO10 | F: CTTGTGTCTTCCTGACCTTGGC  R: CAGTTGGGATGTCGGCACTC | SNP, Expression, qRT-PCR | 55°C | 130 |
| *FRMPD1* | F: TGAATCGAGAACCTGTAGTGACTCG  R: CCTTCGACTGGGAACTGTCTTCAC | SNP, Expression | 60°C | 170 |
| *GABRB2* | F: TGGACCCCCATGAAAATATC  R; TTAGTTCACATAGTAAAGCCAATAG | SNP, Expression | 55°C | 347 |
| *G6PD* | F: TATGACAGTCGGGCCTCCTTC  R: GGCAGAGCGAGGTAGAAGAGG | qRT-PCR | 65°C | 95 |
| EN14997 | F: GAAAGTCAGTCAGCCTTGAGC  R: CCCCAAACAGGTAGGATGGTAGTG | SNP, Expression, BAC, qRT-PCR | 60°C | 184 |
| *GMDS* | F: ACTGCTGATGTTGATGGAGTTGG  R: GAGGATAAAACGGGGTCGTCTC | SNP, Expression, BAC, qRT-PCR | 60°C | 157 |
| *HPRT1* | F: AGACACCGGTAAAACAATGAAAACCTT  R: TCGAGGTGTTCTTTTCACCAACAAG | qRT-PCR | 60°C | 100 |
| *IRX1* | F: CCGGACGGCATCCTCAAGTC  R: AGGTGTAGAGTCCGTGGCTGG | Expression, qRT-PCR  BAC | 65°C | 112  148 |
| *JARID2* | F: GAGCTTCCAAAGTAGACCATGTTGTT  R: GAAGCCCAAGACCGAGGATTT  gDNAF: TCAGATTTCCGACCTCTCTAAAAGG  gDNA R: GGGTTTGACTTCTATGCCG | Expression, qRT-PCR  BAC | 55°C | 87  411 |
| *LOX* | F: CGAGTGAAAAACCAAGGCACATC  R: CGAGCAGGTCATAGTGGCTGAAC | Expression, qRT-PCR  BAC | 60°C | 121  1477 |
| EN02294 | F: CCATCCTGTGGCTCAAAGACAA  R: TGGACATCATCATCGTGGAAAGC | Expression, qRT-PCR,  BAC | 55°C | 175  1873 |
| Ox_plat_124086 | F: GCTGTTCAATTAACCCCCCG  R: GTGTCAGCCACGAAGAATCTCTG | Expression, BAC, qRT-PCR | 55°C | 107  107 |
| *SEMA6A* | F: GCAGAATGAAGGGAAAGCATAAGG  R: GGGTCCAGGGTATCCATCTTGTAG  gDNA R: CTCCATCTACCCCAAAACTC | Expression, qRT-PCR,  BAC | 55°C | 142  442 |
| *SHB* | F: CAAGAAAGCGTGAAGTCCCAAC  R: TGCCAATGCTGGAATCGTAAC  qF: GCCTGTCTCTGGAAGGTGAGG  qR: GGAATAAAGTTACGATTCCAGCATTGG | SNP, Expression, BAC  qRT-PCR | 55°C  65°C | 204  87 |
| *SLC1A1* | F: TTGATAGGGTGGGCAGCACG  R: GCTTCAGTTCCTCACGCTTGG  F: GTCCCAGGAAGCAGATGAAATTGAT  R: CTGTACGAGGTTCTCTGGGAACATA | Expression, BAC  qRT-PCR | 60°C | 132  5796 |
| *SLC6A7* | F: GGTTCTCCTCCAGGGACGGAC  R: ATGGTGGTCGCCGTCCTCAG  gDNA R: TAGGTGTCAGGTGAAAGCG | Expression, qRT-PCR  BAC | 65°C | 100  527 |
| *SYNPO* | F: CGGAGAATGGAGGACAACTCG  R: AGCTGGGTAATGTCTGCTTGAGAC | SNP, Expression | 55°C | 722 |
| *ZNF474* | F: ATCCAACACCACCAAACAGAGCG  R: AGAACCTGGGGACGAAACAGAGAG | Expression, BAC, qRT-PCR | 55°C | 84 |
