# Supplementary material for: The Status of Dosage Compensation in the Multiple X Chromosomes of the Platypus
Source: PLoS Genet. 2008 Jul 25;4(7):e1000140. doi: 10.1371/journal.pgen.1000140 (PMC2453332; doi:10.1371/journal.pgen.1000140)
Supplement: Table S5 — Primers used for allele-specific real-time PCR. (0.03 MB DOC) [file pgen.1000140.s007.doc]

Table S5: Primers used for allele-specific real-time PCR

| Gene | Primer Sequence | Annealing Temperature | Length (bp) |
| --- | --- | --- | --- |
| FBXO10 | F: TCTTAGCCTACTTGTGTCTTCC  R_A: CACCTGGTATTATCTAGGCTGAT  R_C: CACCTGGTATTATCTAGGCTGAG | 60°C | 94 |
| EN14997 | F: TCAGTCAGCCTTGAGCGTG  R_G: ATTTGAAACACTGTTGCTGGACC  R_T: ATTTGAAACACTGTTGCTGGACA | 50°C | 154 |
| GMDS | F_C: GGAGTTGGCACTTTACGGCTTC  F_T: GGAGTTGGCACTTTACGGCTTT  R: GTCTCCCTTTGTGGTATTTCTTGC | 60°C | 125 |
| SHB | F: GCAAGAAAGCGTGAAGTCC  R_A: CCAAGGTTGATCGTATTCATCT  R_G: CCAAGGTTGATCGTATTCATCC | 50°C | 172 |
