# Supplementary material for: The Status of Dosage Compensation in the Multiple X Chromosomes of the Platypus
Source: PLoS Genet. 2008 Jul 25;4(7):e1000140. doi: 10.1371/journal.pgen.1000140 (PMC2453332; doi:10.1371/journal.pgen.1000140)
Supplement: Supplementary file 6 [file pgen.1000140.s004.doc]

Table S2: RNA-FISH dataset

All nuclei that did not have 2 DNA FISH signals (or one in males for X-specific genes) have been removed from the data set to give the results shown below

	**Female**				**Male**			
**Genes**	**No signal**	**1 signal**	**2 signals**	**Total**	**No signal**	**1 signal**	**2 signals**	**Total**
CRIM1	2.0	60.0	22.0	84	0.0	66.0	16.0	82
%	2.4	71.4	26.2		0.0	80.5	19.5	
CAMK2A,SLC6A7,CDX1, Novel1	0.0	22.0	74.0	96	0.0	32.0	68.0	100
%	0.0	22.9	77.1		0.0	32.0	68.0	
Ox-plat_124086	1.0	38.0	44.0	83	2.0	83.0	0.0	85
%	1.2	45.8	53.0		2.4	97.6	0.0	
JARID2, DNTBP1	2.0	15.0	69.0	86	0.0	7.0	84.0	91
%	2.3	17.5	80.2		0.0	7.7	92.3	
GMDS	0.0	15.0	79.0	94	4.0	4.0	64.0	72
%	0.0	16.0	84.0		5.6	5.6	88.9	
IRX1	0.0	13.0	67.0	80	7.0	22.0	48.0	77
%	0.0	16.2	83.8		9.1	28.6	62.3	
APC	0.0	39.0	43.0	82	2.0	74.0	0.0	76
%	0.0	47.6	52.4		2.6	97.4	0.0	
SEMA6A	4.0	48.0	13.0	65	1.0	73.0	0.0	74
%	6.2	73.8	20.0		1.4	98.6	0.0	
ZNF474, LOX	3.0	42.0	34.0	79	1.0	83.0	0.0	84
%	3.8	53.2	43.0		1.2	98.8	0.0	
DMRT2	4.0	41.0	43.0	88	2.0	82.0	0.0	84
%	4.5	46.6	48.9		2.4	97.6	0.0	
SHB	0.0	43.0	42.0	85	8.0	72.0	0.0	80
%	0.0	50.6	49.4		10.0	90.0	0.0	
ENSOANG00000007573	3.0	41.0	47.0	91	1.0	74.0	0.0	75
%	3.3	45.1	51.6		1.3	98.7	0.0	
SLC1A1	0.0	47.0	30.0	77	1.0	86.0	0.0	87
%	0.0	61.0	39.0		1.1	98.9	0.0	
ENSOANG00000014997	0.0	36.0	31.0	67	1.0	74.0	0.0	75
%	0.0	53.7	46.3		1.3	98.7	0.0	
HPRT	0.0	3.0	83.0	86	0.0	4.0	85.0	89
%	0.0	3.5	96.5		0.0	4.5	95.5	
