# Supplementary material for: The Status of Dosage Compensation in the Multiple X Chromosomes of the Platypus
Source: PLoS Genet. 2008 Jul 25;4(7):e1000140. doi: 10.1371/journal.pgen.1000140 (PMC2453332; doi:10.1371/journal.pgen.1000140)
Supplement: Supplementary file 7 [file pgen.1000140.s005.doc]

Table S3: RNA-FISH results for three additional female and one male cell lines
All nuclei that did not have 2 DNA FISH signals (or one in males for X-specific genes) have been removed from the data set to give the results shown below

X-specific

SEMA6A

	No signal	1 signal	2 signals	Total
Female 1	0.0	50.0	30.0	80
%	0.0	62.5	37.5	
Female 2	1.0	41.0	43.0	85
%	1.2	48.2	50.6	
Female 3	1.0	52.0	38.0	91
%	1.1	57.1	41.8	
Male 2	2.0	84.0	0.0	86
%	2.3	97.7	0.0	

DMRT2

	No signal	1 signal	2 signals	Total
Female 1	0.0	37.0	55.0	92
%	0.0	40.2	59.8	
Female 2	0.0	49.0	40.0	89
%	0.0	55.1	44.9	
Female 3	3.0	35.0	37.0	75
%	4.0	46.7	49.3	
Male 2	1.0	97.0	0.0	98
%	1.0	99.0	0.0	

Pseudoautosomal

CRIM1

	No signal	1 signal	2 signals	Total
Female 1	1.0	34.0	42.0	77
%	1.3	44.2	54.5	
Female 2	0.0	47.0	33.0	80
%	0.0	58.8	41.3	
Female 3	0.0	74.0	23.0	97
%	0.0	76.3	23.7	
Male 2	2.0	65.0	21.0	88
%	2.2	73.9	23.9	

GMDS

	No signal	1 signal	2 signals	Total
Female 1	0.0	9.0	84.0	93
%	0.0	9.7	90.3	
Female 2	1.0	12.0	71.0	84
%	1.2	14.3	84.5	
Female 3	0.0	10.0	85.0	95
%	0.0	10.5	89.5	
Male 2	2.0	10.0	71.0	83
%	2.4	12.0	85.6	

Autosomal

HPRT

	No signal	1 signal	2 signals	Total
Female 1	1.0	8.0	78.0	87
%	1.1	9.2	89.7	
Female 2	1.0	12.0	65.0	78
%	1.3	15.4	83.3	
Female 3	3.0	10.0	77.0	90
%	3.3	11.1	85.6	
Male 2	1.0	10.0	78.0	89
%	1.1	11.2	86.7	
